# Design of ProjectRun21: a 14-week prospective cohort study of the influence of running experience and running pace on running-related injury in half-marathoners

**DOI:** 10.1186/s40621-017-0124-9

**Published:** 2017-11-06

**Authors:** Camma Damsted, Erik Thorlund Parner, Henrik Sørensen, Laurent Malisoux, Rasmus Oestergaard Nielsen

**Affiliations:** 1 Section of Sport Science, Department of Public Health, Aarhus University, Dalgas Avenue 4, 8000 Aarhus C, DK Denmark; 2 Section of Biostatistics, Department of Public Health, Aarhus University, 8000 Aarhus, DK Denmark; 3 Sports Medicine Research Laboratory, Department of Population Health, Luxembourg Institute of Health, L-1460 Luxembourg, Luxembourg

**Keywords:** Half-marathon, Running schedule, Running-related injury, Load capacity, Training load

## Abstract

**Background:**

Participation in half-marathon has been steeply increasing during the past decade. In line, a vast number of half-marathon running schedules has surfaced. Unfortunately, the injury incidence proportion for half-marathoners has been found to exceed 30% during 1-year follow-up. The majority of running-related injuries are suggested to develop as overuse injuries, which leads to injury if the cumulative training load over one or more training sessions exceeds the runners’ load capacity for adaptive tissue repair. Owing to an increase of load capacity along with adaptive running training, the runners’ running experience and pace abilities can be used as estimates for load capacity. Since no evidence-based knowledge exist of how to plan appropriate half-marathon running schedules considering the level of running experience and running pace, the aim of ProjectRun21 is to investigate the association between running experience or running pace and the risk of running-related injury.

**Methods:**

Healthy runners using Global Positioning System (GPS) watch between 18 and 65 years will be invited to participate in this 14-week prospective cohort study. Runners will be allowed to self-select one of three half-marathon running schedules developed for the study. Running data will be collected objectively by GPS﻿.﻿ Injury will be based on the consensus-based time loss definition by Yamato et al.: “*Running-related (training or competition) musculoskeletal pain in the lower limbs that causes a restriction on or stoppage of running (distance, speed, duration, or training) for at least 7 days or 3 consecutive scheduled training sessions, or that requires the runner to consult a physician or other health professional*”.

Running experience and running pace will be included as primary exposures, while the exposure to running is pre-fixed in the running schedules and thereby conditioned by design. Time-to-event models will be used for analytical purposes.

**Discussion:**

ProjectRun21 will examine if particular subgroups of runners with certain running experiences and running paces seem to sustain more running-related injuries compared with other subgroups of runners. This will enable sport coaches, physiotherapists as well as the runners to evaluate their injury risk of taking up a 14-week running schedule for half-marathon.

## Background

After more than two decades with a steadily increase in the engagement into running (Billat, 2005; Pilgaard & Rask, 2016) the popularity of running now ranks in the top of the most popular physical activities (Pilgaard & Rask, 2016; Cave & Miller, 2016). Further, thanks to its high practical feasibility, running has reached out broadly in a multitude of countries (Billat, 2005; Cave & Miller, 2016; Buist et al., 2010). Historically, the five and 10 km running distances have been the main standard distances, demonstrated by a continually high number of runners participating in race events on these distances (DAF & MotionDANMARK, 2014). Currently, a tendency towards an inversion of this picture seems to be ongoing, with a growing interest towards longer distances such as half-marathon and marathon. Since 2000, a particular attractiveness of running a half-marathon has been steeply increasing (DAF & MotionDANMARK, 2014; Running USA, 2014), showed by a 307% growth in United States half-marathon finishers (from 482,000 in 2000 to 1,960,000 in 2014). In parallel, an increasing number of official half-marathon racing events have been organized (Running USA, 2014). This popularization of half-marathon is also reflected by the surface of a vast number of free half-marathon running schedules that now are available in sports magazines and on the Internet.

Similarly to other forms of physical activity, training for half-marathon has advantageous impacts on health-related factors, including reduced all-cause mortality (Evenson et al., 2016; Lee et al., 2014), increased quality of life (Pedersen & Saltin, 2015), and reduced risk of many chronic- and lifestyle diseases (Pedersen & Saltin, 2015). Furthermore, through its high positive influence on the cardiovascular and respiratory systems (Warburton et al., 2004) it has direct effects on variables like weight and physical fitness (Hespanhol Junior et al., 2015), which both have been indicated to be of great motivation for continued running (Dyrstad and Tjelta 2013).

Unfortunately, all the health effects achievable from training for half-marathon may, however, be offset by the high risk of sustaining a running-related injury (RRI). This is highlighted by (Kluitenberg et al., 2015) who found a pooled injury incidence proportion for long-distance road runners (between 10 km and less than a marathon) on 31.7% during a 1-year follow-up. In addition, RRI has been found to be the main reason for a temporarily or even a permanently stop of running (Koplan and Jones 1995; Forsberg 2014; Kluitenberg et al., 2014).

In the scientific literature, it has been suggested that up to 90% of all RRI’s develop as a consequence of overuse of body tissue (muscles, tendons and bones) (Lysholm & Wiklander, 1987; Nielsen et al., 2014a). Theoretically, overuse injuries occur as a result of a cumulative process of tissue damage (Finch & Cook, 2014; Timpka et al., 2014) that leads to injury if the cumulative training load over one or more training sessions exceeds the runners’ load capacity for adaptive tissue repair (Hreljac, 2005; Soligard et al., 2016). This process is often referred to as “training errors” or as “running too much too soon” (Lysholm & Wiklander, 1987; Hreljac, 2005; Nielsen et al., 2012; Wen, 2007; Johnston et al., 2003; James et al., 1978; McKenzie et al., 1985; Jacobs & Berson, 1986; Buist et al., 2008), and implies that the onset and development of RRI is strongly related to insufficient management of training loads in respect to load capacity (Soligard et al., 2016; Meeusen et al., 2013; Bertelsen et al., 2017; Nielsen et al., 2017). Emerging evidence for this relationship between training load and injury risk is currently developing within sports science in general, and especially sudden changes in the training load is now suggested to play a key role for injury development (Bertelsen et al., 2017; Nielsen et al., 2017; Malisoux et al., 2015; Drew & Finch, 2016; Nielsen et al., 2014b; Hulin et al., 2016; Gabbett et al., 2016). However, owing to intra- and inter-individual variation in e.g. age, sex, BMI, previous injury status, running- experience and pace etc., the magnitude of the training load a runner is able to withstand before the load capacity is exceeded will vary (Bertelsen et al., 2017; Nielsen et al., 2017).

Since training load is highly determined by the exposure to running (Petersen et al., 2015; Schache et al., 2011) the running exposure must, from a injury prevention perspective, be carefully differentiated in respect to customized training adaptations taking into account the load capacity the individual runner possesses prior to being exposed to training loads (Soligard et al., 2016; Meeusen et al., 2013; Meeuwisse et al., 2007).

Therefore, an estimate of load capacity becomes central in relation to planning appropriate running schedules for half-marathon. Several estimates of load capacity have been suggested including both psychological, physiological and other biochemical as well as hormonal and immunological variables (Soligard et al., 2016; Meeusen et al., 2013). However, as occurrence of overuse injuries in runners most likely will be a matter of biomechanical stressors primary related to the ground reaction forces acting on the muscles, tendons and bones at each strike impact, measures of the muscle- and tendon strength and bone density may be more relevant as estimates of the load capacity when speaking RRI (Hreljac, 2005). Due to the time consuming and costly challenges related to direct objective individual assessments of such variables in large epidemiological studies, appropriate surrogate information representing these variables is more beneficial and feasible to obtain (Juul 2004).

Load capacity is modifiable in that way that it can both decrease along with increased inactivity and with insufficient respect to appropriate rest between running sessions, while it is positively modifiable as a result of repeated adaptive running training (Soligard et al., 2016). It is therefore plausible to assume that the level of load capacity can be tipped of by how much running experience and the pace abilities the runners possess prior to running participation. Those two variables can then provide us with a quantitative estimate of how much running a runner is able to tolerate before the limits of the load capacity is reached.

By this approach, runners with lowest running experience (lower average weekly running distance) and/or lowest running pace (slower maximal running pace) will have the lowest load capacity compared with their counterpart runners.

However, evidence-based knowledge about how to differentiate the training load in relation to running experience and running pace while seeking to minimize injury risk does not seem to exist.

The aim of ProjectRun21 is, therefore, to investigate the association between running experience and running pace on the risk of running-related injury amongst runners following the same 14-week running schedule (a distance-based or a pace-based or a mixed of those two) for half-marathon, based on the following hypotheses:

1. Low experienced runners will sustain more injuries compared with high experienced runners after the first 50 km of following a distance-based 14-week running schedule for half-marathon.
2. Low-pace runners will sustain more injuries compared with high-pace runners during the first 50 km of following a pace-based 14-week running schedule for half-marathon.
3. Low-pace runners with a low running experience will sustain more injuries compared with high-pace runners with a high running experience during the first 50 km of following a mixed 14-week running schedule for half-marathon. Furthermore, it is hypothesized that the injury risk due to interaction is higher when running experience and running pace act together in a synergism compared with an addition of their discriminative injury risks.

## Methods

### Study design

ProjectRun21 is designed as an observational prospective cohort study with 14-week follow-up. It consists of three sub-cohorts based on three different pre-developed running schedules for half-marathon (see description of the running schedules below).

#### Research reporting and ethics

All scientific articles of this research will follow the STROBE-statement developed to strengthening the reporting of observational studies in epidemiology (von Elm et al., 2008). The study design and its procedures have been presented to the local ethics committee (record number “request 187/2015”). However, according to the Danish law, the study was not considered for ethical approval, as observational studies do not require ethical approval in Denmark. The Danish data protection agency has approved the study including the data collection procedures (The Danish Data Protection Agency’s journal-number: 2015–57-0002; Aarhus University’s journal-number: 62,908, serial number 224).

All runners have to approve an online-based informed consent by clicking in a checkbox in the baseline questionnaire, in order to be eligible for inclusion. The participants will be allowed to discontinue participation at all times without providing a reason.

The development of the three running schedules is based on already existing running schedules used in practice that targets a broad range of different runners (see detailed description of the development of the running schedules below). Therefore, the risk of injury amongst the included runners will be expected to be equal to the risk of injury amongst runners choosing a similar running schedule on their own from the Internet.

### Study population, recruitment strategy and inclusion

Runners at all levels interested in participating in the study are invited to sign up for participation through an online-based baseline questionnaire.

#### Recruitment strategy

To improve and promote the recruitment of runners, contact will be taken to sports shops, running clubs, sports and health departments in universities, and the news media, as well as through contact to persons with a high number of followers on the social medias. Study-specific recruitment material will be distributed via posts on social medias, through newsletters, handouts of flyers, newspaper articles, radio spots/interviews and others.

In order to make the follow-up period as suitable for as many runners as possible, the runners will be allowed to freely choose which date (any giving Monday in the recruitment period during summer and fall 2016) they prefer to start their 14-week active participation. To improve compliance to the running schedule, the runners are allowed to self-select between one of the three schedules developed for this study.

#### Inclusion in the study

After fulfillment and submission of the baseline questionnaire, all runners will be screened for eligibility to participate, which implies fulfillment of the following inclusion criteria:

1. 18 years or above.
2. Agree to follow one of the available running schedules.
3. Agree to use a GPS-watch or an application for Android- or iOS-based smart-phone to quantify their running.
4. Agree to report running data if any, via daily e-mails
5. Agree to fill out e-mail-based weekly questionnaires covering injury status, health status, use of the health-care system, changes in weight, participation in other sports, and other supplemental questions.
6. All participants must approve an informed consent form before inclusion in the project.

Persons will be excluded if they:

1. have had a RRI in the lower extremity or lower back 6 months preceding baseline,
2. and/or have had any other injury limiting their intended running activity the past 6 month,
3. and/or if any contraindications for vigorous physical activity are present: Symptoms of heart or chest pain, previous heart or chest surgery, lung diseases, dizziness or discomfort when physically active, pregnancy or non-regulated diabetes.

Runners fulfilling the inclusion criteria will receive a welcome letter by e-mail from the ProjectRun21 mail address (pr21@ph.au.dk), which will include detailed information about the study procedures and the participation.

### Data collection

Running data will be collected objectively through a GPS-watch or an application for Android- or iOS-based smartphones, of which the brand and model are free of choice, as long as it is able to measure running distance (kilometers), duration (time), and pace (minutes/kilometers).

All data will be uploaded to an Internet-based personal diary developed by Help2Run (http://www.mit-loebeprogram.dk). Data storage will be hosted by Amazon and backed-up by a Help2Run server placed in Hornslet, Denmark.

The data uploaded to the personal diary will be accessible for the researchers through a SSL-protected back-end system allowing all runners to be under continuous surveillance during follow-up. From the back-end, data can be extracted for data management and statistical analyses.

#### Demographics

In order to establish a in-depth picture of the study population, and thereby, be able to elucidate which target population the results of the current study can be generalized to, all runners have to fulfill a comprehensive baseline questionnaire including a range of different information about their demographics, previous and existing injuries, health and illness status, use of the health-care system, how many years they have been running since the age of 18 (>2 years is reported in years, otherwise in months), their volume of running within the past six months (typical weekly running distance, shortest and longest running distance, participation in any running competitions or achievements of any personal records), running style, running equipment (shoes and orthotics), and hours of participation in other sports. Subsequently, they have to choose the start date and the running schedule they wish to follow, and finally, they need to approve informed consent for participating as outlined in the information material.

#### Running data

On a daily basis, an automated e-mail including a link to a short questionnaire about running participation on that particular day will be distributed to all participants. A positive answer to the first question “I have been running today” opens up for additional answer fields to report the distance, the duration, and the pace. Furthermore, fields for reporting the intensity (measured subjectively using the CR-10 scale (Borg, 1982)), if they felt able to continue running at the same pace (if “yes”, how far?), and if they followed the running schedule as prescribed (if “no”, they can report how their training differed) will appear.

In case of no submission of the daily training questionnaire, a reminder e-mail will be send the day after in order to remind the runners to report their running data.

#### RRI, health and illness data and data on co-variants

On a weekly basis, another automated e-mail containing a link to questions regarding injury, health, illness and supplemental questions will be distributed throughout the follow-up period. As in the daily e-mails, a positive answer to the initial question expands the questionnaire with detailed questions about injury as well as about health and illness status. The supplemental questions will cover weight change, change in the amount of participation in other types of sports, and other relevant supplemental information. Finally, they will have the opportunity (voluntary) to report reasons for abstain from following the pre-developed running schedule or for fully discontinuation of running.

### Outcome

The outcome of interest is RRI, which will be classified based on the consensus-based dichotomized injury definition (injury yes/no) recently developed by (Yamato et al., 2015): “*Running-related (training or competition) musculoskeletal pain in the lower limbs that causes a restriction on or stoppage of running (distance, speed, duration, or training) for at least 7 days or 3 consecutive scheduled training sessions, or that requires the runner to consult a physician or other health professional*”.

Collection of RRI data, health and illness status, and data on co-variants will be based on subjective answers to a modified version the Oslo Sports Trauma Research Center (OSTRC) Overuse Injury Questionnaire, the OSTRC health and illness questionnaire, and supplementary questions, respectively. The OSTRC is developed and validated by (Clarsen et al., 2013), and subsequently translated into- and validated in Danish by (Jorgensen et al., 2015).

Modification of the OSTRC questionnaire by inclusion of an additional question related to each anatomical location is needed in order to cover if the injury fulfills the injury definition by (Yamato et al., 2015) The following question will be added: “Have your problems with your (foot, ankle, lower leg, knee, groin, hip, buttock, or lower back) restricted your running activity (distance, speed, duration, or training) for at least 7 days or 3 consecutive scheduled training sessions, or that requires the runner to consult a physician or other health professional”. The OSTRC questionnaire enables for recording broad aspect of injury consequences including injury status, pain, time-loss from training and competition, reduced performance and medical attention. This information may be used for secondary analysis of hypothesis related to those variables.

### Exposure

Running experience will be the primary exposure for the cohort following the distance-based schedule, while running pace will be the primary exposure for the cohort following the pace-based schedule. For the cohort following the mixed schedule, both the running experience and running pace will be the exposure. Thus, in the analyses the association between running experience and injury (data from the distance based schedule), and between running pace and injury (data from the pace-based schedule), as well as their interaction (data from the mixed schedule), will be investigated independently through the three different running schedules for half-marathon.

#### Running experience

Running experience will be quantified in kilometers on a continuous scale, and will be assessed through answers to the baseline questionnaire about how many kilometers the runners typically have been running per week on over the past 6 months prior to inclusion. Since information about running experience is assessed at baseline, running experience prior to the study will be included in the analyses as a time-fixed exposure.

Owing to the plausible likelihood of a potential higher injury risk in line with decreased running experience, the relationship between running experience and RRI is believed to be non-linear. Running experience will therefore be included in the analyses as a dichotomized exposure split into a high and a low group with a cut-off value on 15 km per week. The chosen pre-fixed cut-off values is set a priory data collection based on two previous studies collecting data of the average running distance per week from 925 novice and recreational runners (553 participants in one study and 372 participants in the other study) (Malisoux et al., 2016a; Malisoux et al., 2016b).

#### Running pace

**Fig. 1:**
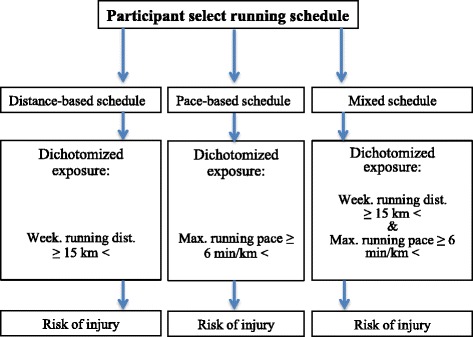
An overview over the exposure for each running schedule. The risk of injury is reported for each running schedule independently. Min = minutes. Km = kilometers. Week. = weekly. Dist. = distance. Max. = maximal

Running pace is assessed through a 2 km start-test embedding in the running schedules on week one, training session two. Running pace will then be determined on a continuous scale using the average running pace (minutes per kilometers) obtained during the start-test. Similarly to running experience, the running pace will only be assessed at baseline, and will therefore also be considered as a time-fixed variable in the analyses. Further, a non-linear potential relationship is equally believed to exist for the relationship between running pace and RRI, and thus, running pace will also be analyzed as a dichotomized exposure split into a high and a low group. The cut-off value dichotomizing the running pace is set to 6 min/km, which in line with the cut-off for the running experience is chosen a priory data collection based on two previous studies collecting data of the average running pace (Malisoux et al., 2016a; Malisoux et al., 2016b). (See Fig. 1).

### Running schedules

#### Development

Three different running schedules for half-marathon have been specifically developed for the present study. Each of them focuses on investigating either the effect of 1) running experience in the cohort of runners following the distance-based schedule, 2) running pace in the cohort of runners following the pace-based scheduled or 3) the interaction between those two variables in the cohort of runners following the mixed schedule, on the risk of RRI. All of them have been developed by the former Danish national coach within middle- and long distance running with emphasis on mirroring the running schedules being used in practice. Common for the three schedules are their duration of 14 weeks and the session frequency of three running sessions per week, summed up to a total of 42 sessions in each schedule. The differences in the design of the three pre-developed schedules are shown in Table 1.

**Table 1:** An overview over the differences between the pre-designed running schedules

	The distance-based schedule	The pace-based schedule	The mixed schedule
Exposure	Running experience	Running pace	Running experience and running pace
Total km	363 (hereof easy 363)	257 (hereof easy 203,7)	295,1 (hereof easy 274,6)
Km per week	25,9	18,4	21,1
Number of sessions	42	42	42
Distance per session	8,6	6,1	7
Intensive km (excl. Halfmarathon)	0	53,3 (submax 36,3, max 17)	20,5 (submax 10,5, max 10)
Intensive sessions (excl. Halfmaraton)	0	20	7
% intensive km	0%	21%	7%
Total expected training time in min.	1997	1378	1608
Expected training time per week in min.	142,6	98,5	114,8
Training time at:			
- Easy	1997	1120	1510
- Submax	0	182	53
- Max	0		45

legend: Km = kilometers. Excl. = Exclusive. Submax = submaximal. Max = maximal. Min. = minutes

The clues for the running pace are analogously for all schedules (see legends to Tables 2, 3 and 4). Where no pace is indicated the session should be run in the preferred and natural pace.

**Table 2:** The distance-based schedule

Week no.	Day 1	Day 2	Day 3
1	5 km	START-TEST 1 km easy, 1 km preferred, 2 km fastest possible*, 1 km easy	8 km
2	5 km	5 km	9 km
3	5 km	5 km	10 km
4	5 km	5 km	11 km
5	5 km	6 km	12 km
6	5 km	6 km	13 km
7	5 km	6 km	14 km
8	6 km	6 km	15 km
9	5 km	8 km	16 km
10	5 km	9 km	17 km
11	5 km	10 km	18 km
12	5 km	10 km	15 km
13	10 km	FINAL-TEST 1 km easy, 1 km preferred, 2 km fastest possible*, 1 km easy	12 km
14	8 km	4 km	Half-marathon 21,1 km

legend: No. = number. Km = kilometers. Easy = Slightly slower pace than the preferred and natural pace, and should be completed consciously slowly with surplus energy. Preferred = The preferred and natural pace. Fastest possible = The highest pace as possibly in relation to the distance. * = The star indicates that 1–3 min. Break is allowed after the 2 km run in running pace “fastest possible”. The GPS-unit has to be paused during breaks

**Table 3:** The pace-based schedule

Week no.	Day 1	Day 2	Day 3
1	5 km	START-TEST 1 km easy, 1 km preferred, 2 km fastest possible*, 1 km easy	5 km
2	1 km easy, 2 km fast, 1 km easy	4 km	2 km easy, then 3 km switching between 200 m fast and 100 m easy
3	4 km	1 km easy, 3 km fast, 1 km easy	8 km
4	1 km easy, 2 km fast, 1 km easy	5 km	1 km easy, 1 km preferred, 1 km fast, 1 km fastest possible*, 1 km easy
5	4 km	2 ½ km easy, 2 ½ km fastest possible	9 km
6	1 km easy, 2 km fast, 1 km easy	4 km	2 km easy, then 4 km switching between 300 m fast and 200 m easy
7	5 km	1 km easy, 1 km preferred, 1 km fast, 1 km fastest possible*, 1 km easy	10 km
8	1 km easy, 2 km fast, 1 km easy	5 km	2 km easy, 3 × 1 km fastest possible*, 1 km easy
9	4 km	2 km easy, 4 km fast, 1 km easy	10 km
10	1 km easy, 2 km fast, 1 km easy	5 km	3 km easy, 4 km fast, 3 km easy
11	4 km	2 ½ km easy, 2 ½ km fastest possible	12 km
12	1 km easy, 2 km fast, 1 km easy	5 km	2 km easy, 5 km fast, 2 km easy
13	7 km	FINAL-TEST 1 km easy, 1 km preferred, 2 km fastest possible*, 1 km easy	12 km
14	2 km easy, 3 km fastest possible*, 1 km easy	4 km	Half-marathon 21,1 km

legend: No. = number. Km = kilometers. Easy = Slightly slower pace than the normal and natural pace, and should be completed consciously slowly with surplus energy. Preferred = The preferred and natural pace. Fast = Faster pace than preferred and natural pace, but slower than fastest possible allowing for further running afterwards. Fastest possible = The highest pace as possibly in relation to the distance. * = The star indicates that 1–3 min. Break is allowed after the 2 km run in running pace “fastest possible”. The GPS-unit has to be paused during breaks

**Table 4:** The mixed schedule

Week no.	Day 1	Day 2	Day 3
1	5 km	START-TEST 1 km easy, 1 km preferred, 2 km fastest possible*, 1 km easy	6 km
2	5 km	5 km	7 km
3	5 km	1 km easy, 3 km fast, 1 km easy	7 km
4	5 km	5 km	8 km
5	6 km	2 ½ km easy, 2 ½ km fastest possible	8 km
6	5 km	5 km	9 km
7	5 km	1 km easy, 1 km preferred, 1 km fast, 1 km fastest possible*, 1 km easy	10 km
8	5 km	6 km	11 km
9	5 km	2 km easy, 4 km fast, 1 km easy	12 km
10	5 km	6 km	13 km
11	5 km	2 ½ km easy, 2 ½ km fastest possible	15 km
12	5 km	6 km	13 km
13	7 km	FINAL-TEST 1 km easy, 1 km preferred, 2 km fastest possible*, 1 km easy	15 km
14	5 km	4 km	Half-marathon 21,1 km

legend: No. = number. Km = kilometers. Easy = Slightly slower pace than the normal and natural pace, and should be completed consciously slowly with surplus energy. Preferred = The preferred and natural pace. Fast = Faster pace than preferred and natural pace, but slower than fastest possible allowing for further running afterwards. Fastest possible = The highest pace as possibly in relation to the distance. * = The star indicates that 1–3 min. Break is allowed after the 2 km run in running pace “fastest possible”. The GPS-unit has to be paused during breaks

#### The distance-based schedule

The distance-based schedule is developed with specific focus on preparing the runner for half-marathon by increasing the weekly running distance throughout the 14 weeks. No interval training sessions are included in this running schedule. Instead, the runners have to run all sessions at preferred pace (rather a little slower than too fast) (see Table 2).

#### The pace-based schedule

The pace-based schedule is developed with emphasis on interval training in two out of three of the training sessions. In order to equalize the running intensity among all runners engaging into this running schedule, the following four-point intensity-scale based on clues of perceived exhaustion will be used to guide the running intensity in the interval sessions: 1 = Easy, slightly slower pace than the preferred and natural pace, and should be completed consciously slowly with surplus energy. 2 = Preferred, the preferred and natural pace. 3 = Fast, faster pace than preferred and natural pace, but slower than fastest possible allowing for further running afterwards. 4 = Fastest possible, the highest pace as possibly in relation to the distance. In the remaining one third of the sessions, the runners are prescribed to run a distance-based session at preferred pace (rather a little slower than too fast) (See Table 3).

#### The mixed schedule

Combining running distance and running pace, the mixed schedule is developed with focus on increasing both the weekly running distance (to a lesser degree than in the distance-based-schedule) and incorporating sessions with interval training (fewer and shorter than in the pace-based schedule). In one out of three weekly training sessions, interval training will be in focus. In the other two third of the weekly training sessions, the runners are prescribed to distance-based running at preferred pace (rather a little slower than too fast) (See Table 4).

### Statistics

Time-to-event statistics (pseudo-observation method through a generalized linear regression model) will be utilized to analyze the association between running experience or running pace and the risk of RRI after 50 km of follow-up. Runners will be censored in case of: discontinuation of the running schedule due to lack of motivation and/or time, health- and illness problems, and other personal concerns hindering further participation.

Cumulative risk difference will be used as measure of association (Parner & Andersen, 2010). The cumulative risk differences between time-to-first-injury will be compared between groups within each exposure using kilometers (primary analyses), weeks and training sessions as time-scales. Data will be analyzed at the following points using kilometers as time-scale: 50, 100, 200 km and by the end of follow-up, which is at 363, 257, and 295 km for the distance-based, page-based and mixed schedule respectively. Using weeks as time-scale, data will be analyzed at 2, 4, 8 and 14 weeks, and with training sessions as time-scale, data will be analyzes after 6, 12, 24 and 42 sessions.

Competing risk analysis, using the Aalen-Johansen estimator, will be performed separately for both exposures, in order to take into account that runners also can sustain injuries from other sports competing to RRI during follow-up (Putter et al., 2007). If a runner is injured due to participating in another sport, they will no longer be at risk of sustaining a RRI because their scheduled running has been interrupted by this competing event. Therefore, these runners should not be included in the analyses as still contributing to the probability of sustaining a RRI (Putter et al., 2007).

The primary analysis will be run based on the assumption that the runners are compliant to their selected running schedule. Results will be presented with estimated precision (95% confidence interval), and will be considered statistically significant at *P* < 0.05. All statistical analysis will be conducted using STATA version 12 or greater.

To investigate if an interaction exist between running experience and running pace in such way, that a synergism of those two exposures is greater than the sum of the effects of each exposure separately, the absolute excess risk due to interaction will be calculated on an additive scale (Knol & VanderWeele, 2012). As for the estimation of the association between running experience and running pace on the risk of RRI, results for the interaction will be presented with 95% CI and *P*-values.

To study if the association between running experience or running pace and the risk of RRI may be modified, and thereby differ across strata of different demographics and other types of sports activities; BMI, age, previous injury, and participation in other sports activities will be included in complementary risk factor analyses as effect-measure modifiers in accordance with the recommendations by (Rothman et al., 1980). Stratified analyses according to BMI, age, previous injury and participation in other sports activities will be conducted. All results of the stratified analyses will be presented as the cumulative risk differences with 95% CI between exposure groups and within strata of each effect-measure modifier. When using the pseudo-observation method to estimating risk differences in stratified analyses examining sample sizes of 50 and above, (Hansen et al., 2014) have found that at least 10 events (injuries) are needed per variable (modifier) in order to avoid violation of the statistical assumptions for valid analysis. Inclusion of any modifying variable will be determined in accordance with the recommendations described by (Hansen et al., 2014).

#### Power calculation

Using a superiority model, power has been calculated independently for the two time-fixed exposures: running experience and running pace. Based on previously collected data on high (long-distance runners) (Rasmussen et al., 2013; Hirschmüller et al., 2012) and low (novice) experienced runners (Nielsen et al., 2014c; Van Ginckel et al., 2009), an injury incidence of 12% is expected for high experienced/high-pace runners, while 40% for their counterpart peers. To be able to show a minimum difference of 5% in injury risk between groups of running experience and 3% between groups of running pace, a sample size of respectively 110 runners (50 low experienced and 60 high experienced) and of 86 runners (51 low-pace and 35 high-pace) is needed in order to reach a desired power of 80%.

However, according to two previous prospective cohort studies within RRI research it is necessary to take into account a 14–22% potential loss to follow-up when determining the number of participants needed to include in the study (Malisoux et al., 2016a; Nielsen et al., 2013a). Therefore, a loss to follow-up on 20% will be added to the sample sizes equivalent to 138 participants for the investigation of the association between running experience and RRI risk, while 108 participants for the association between running pace and RRI risk.

## Discussion

ProjectRun21 will be the first study to prospectively include running experience and running pace as primary exposures for development of RRI while following a specific running schedule for half-marathon. With this knowledge lights will be shed upon if particular subgroups of runners seems to sustain more RRI compared with other subgroups of runners. Owing to the high popularity of engaging into half-marathon running, the results will be highly valuable for both runners as well as sport coaches and physiotherapists in practice to evaluate runners’ injury risk and thereby get an indication of their readiness of taking up 14-weeks of half-marathon training. Furthermore, for the runners, the results will add to their own assessment of their load capacity for engaging into a half-marathon running schedule available in sports magazines and on the Internet.

In the present study it is hypothesized that runners with a low running experience and/or a low running pace at baseline will be more prone for sustaining a RRI compared with their counterpart runners. Confirmation of these hypotheses may indicate that less experienced and/or runners with a low running pace may benefit of performing more pre-conditioning exercises or progressive introduction into running before entering and/or during a specific running schedule for half-marathon. However, due to the observational design of the current study, data only allows for an overview of the injury risk for different subgroups of runners, while it does not provide detailed recommendations of how to schedule and progress the half-marathon training load for different runners in relation to prevention of RRI.

Although general agreement exist supporting the risk of running too much too soon, and despite emerging evidence is developing for the relationship between training load and injury risk, knowledge of how to progress the half-marathon training load in relation to load capacity is still scientifically sparsely explored. Therefore, in order to address this limited knowledge, a self-structured running schedule for half-marathon with no requirements to training load will be included in the study. The purpose of including the self-structured running schedule is to investigate the association between different progressions in the training load and RRI for different runners, and thereby shed light on potential injurious sudden changes in training load. Therefore, the primary exposure for the self-structured running schedule will be the change in training load. Compared to the time-fixed exposures: running experience and running pace, changes in training load differs in that way that it constantly varies over time, and therefore, will be included as a time-varying variable in the analyses.

In relation to the time-fixed exposures, the influence of running experience (defined in various ways) has previously been investigated, although these studies fails to show any uniquely results (Hulme et al., 2017). These diverse findings may mainly be explained by: 1) that no consensus exists about how to define running experience or of the exact nomenclature of different runners (i.e. novice and recreational runners). 2) that the training load during the observation period has varied between participants without this being taking into account in the analyses.

According to the first, running experience has typically been measured as the cumulative monthly or yearly units of running (Hulme et al., 2017), which allows for large variations in the quantification of kilometers different runners actually have been running. Hence, two runners with the same monthly (or yearly) experience may likely be regarded as having the same running experience despite potential high differences in completed kilometers. However, with the training load playing a key role for RRI development, the number of completed kilometers becomes an essential variable that must be included in the definition of running experience. Furthermore, with no established consensus of how to define different runners, runners may be misclassified to a specific running population, which yet may add to the conflicting results.

Regarding the latter; seen from a causal perspective, exposure to training load is the only necessary cause for development of RRI, since no RRI is able to occur without engaging into running practice. Therefore, the exposure to training load must be strictly accounted for in the analyses when investigating risk factors for RRI (Malisoux et al., 2015). In relation to the aim of the current study investigating the association between the running experience and risk of injury, it will not be possible to differentiate if injury development is due to different exposure of running training load or to the running experience prior to baseline if the level of running participating is not corrected for.

Contrasting results have also been reported in relation to the influence of running pace on RRI (Hulme et al., 2017). Inconsistency in definition and/or how to measure running pace such as subjectively reports of Ratings of Perceived Exertion (RPE), or objectively as speed in kilometers per hour (km/h) combined with unequal exposure to this variable between participants, may explain the obscurity related to the knowledge of this association.

One of the specificities and strengths of the current study is that running experience and running pace are included as the primary exposures, while the exposure to running is conditioned by design. Using a fixed running schedule for half-marathon and thereby, a fixed exposure to running as in our protocol, is a tremendous asset to investigate the influence of running experience and running pace on RRI risk, as a potential confounding effect of different exposure to running will be eliminated. Further, this study design opens the door to the inclusion of other co-variants (BMI, previous injury, age, and participation in other sports activities) as effect-measure modifiers allowing for detailed information of the RRI risk in different subgroups of runners.

Another strength is the applied data collection method, as the use of GPS recently has been validated as an objective measure of running participation both in relation to quantification of running distance in kilometers (Nielsen et al., 2013b) and of running speed (Townshend et al., 2008).

However, a major limitation is related to the unawareness of how compliant the participants will be to the running schedule. If the compliancy will be low, the fixed training load encompassed in the running schedule will death to be equal to all participants, which then will be able to affect the association between running experience or running pace and the risk of RRI.

In the current study it is believed that a non-linear relationship exist between the exposures and injury development in that way that the injury risk is potentially increasing in line with decreased running experience. If such non-linearity exist, the statistical assumptions for including the exposures as continuous variables in the analyses will not be fulfilled, which has led us to dichotomize the exposures as described in the methods section. One concern is related to the accuracy of the pre-fixed cut-off values chosen for this dichotomization, since they are based on previously data collection including 925 novice and recreational runners (Malisoux et al., 2016a; Malisoux et al., 2016b) following a non-fixed running regime with no requirement to complete a half-marathon by the end of follow-up. Thus, data used to establish the cut-off values are based on a study population that distinguish from the one in the current protocol, which may constrain the relevance of the chosen cut-offs. However, if the relationship between the exposures and RRI turns out to be linear related, inclusion of the exposures as continuous variables will be considered in the time-to-event analyses rather than using the dichotomized cut-off values.

Possible limitations related to bias as information bias and selection bias may occur in the present study, which for the first relates to the self-reporting of injury status and for the second to the free choice of running schedule. As to avoid information bias related to subjective reports and evaluations of RRI, comprehensive clinical examinations of these injuries may be preferably in large epidemiological studies to accurately assess the relationship between exposure and outcome (in this case RRI). However, the OSTRC questionnaires will be distributed on a weekly basis, and will provide detailed information about the onset and development of injury throughout follow-up. This combined with no need for specific injury diagnoses; it is believed that the injury status will be reported with a sufficient accuracy. Regarding selection bias, the primary concern is related to the study design allowing participants to choose their own running schedule. This may lead to an underestimation of the injury risk in such way, that the participants are able to choose the kind of running schedule they are most familiar and confident with, and which therefore, may be less injurious for that specific runner compared to other running schedules. On the other hand, this approach may facilitate the runners’ compliance to the chosen running schedule compared to random allocation to a particular running schedule, which they might not feel motivated for completing.

## Abbreviations

GPS: Global Positioning System
OSTRC: the Oslo Sports Trauma Research Center
RRI: Running-related injuries
